# Life on Venus?

**DOI:** 10.3390/life15050717

**Published:** 2025-04-29

**Authors:** Sanjay S. Limaye

**Affiliations:** Space Science and Engineering Center, University of Wisconsin-Madison, Madison, WI 53706, USA; sslimaye@wisc.edu; Tel.: +1-608-262-9541

**Keywords:** Venus, life, phosphine, ammonia, habitability, microorganisms

## Abstract

Venus is not generally at the forefront when considering extraterrestrial life. Yet, based on the physical similarities and proximity to Earth and with the little knowledge of its evolutionary history, there is a possibility that Venus may have hosted life in the past on the surface if Venus had liquid water and perhaps even has water present in the clouds today. While the early suggestions during the beginning of the space exploration about life on Venus were mostly speculative due to limited data, recent interest has arisen from realizations: (i) the unexplained ultraviolet absorption spectrum of Venus resembles many organics, (ii) there is chemical disequilibria in the cloud layer, (iii) the cloud aerosols likely contain significant abundances of hydrated iron and magnesium sulfates, and (iv) the solar radiation received in the cloud layer contains the appropriate wavelengths and flux to support phototrophy. Considering the extreme environmental survival of many terrestrial microorganisms, the possibility remains that any extant life on Venus in the past could have adapted to survival in the cloud layer far above the surface where energy and nutrients are available, but the precise compositions of the cloud particles and water availability are still uncertain. The key to solving the mystery of life on Venus is to determine if Venus had liquid water on the surface in its past and to measure the precise chemical composition of the Venus atmosphere and the cloud particles. Missions which will be launched in the next few years will provide much needed data that should provide some answers we seek and will surely raise more questions. This perspective reviews recent developments.

## 1. Introduction

More than a century ago when few properties of the Venus atmosphere were known, Arrhenius [1] speculated that Venus had plenty of liquid water with forests and flora and fauna. In the following five decades more was learned about the atmosphere and surface through ground-based and balloon telescopes and first space missions. These led to the first suggestion of life in the clouds of Venus [2] based on the speculation that the cloud particles were water based.

Measurements from atmospheric entry probes, fly-by spacecraft, orbiters, and balloons floating in the Venus atmosphere (1967–1985) revealed that the mostly carbon dioxide atmosphere and the surface were quite warm and devoid of liquid water and harbored clouds of concentrated sulfuric acid [3]. The realization that the surface and perhaps even the clouds were far different from early speculations quickly dispelled any notions of extant life.

The early discoveries about Venus and its atmospheric superrotation [3,4,5,6,7] included some mysterious ones that could be interpreted as possible presence of life. Boyer [8] following a suggestion by his friend Paul Malaval, invoked microorganisms, or “biotes” to explain the acceleration of the atmospheric flow from morning to noon through solar energy absorption. Others included chemical disequilibrium [9,10,11], absorptive properties [12,13], and the possible past presence of liquid water [14]. Specifically, the possible presence of ammonia [9] and molecular oxygen was puzzling. Discovery of ammonia was quickly dismissed as it could be present under equilibrium conditions [15], which may have hindered future measurements and progress. One major mystery that remains today is the nature and identity of the ultraviolet absorbers in the clouds [16,17,18,19,20,21]. The persisting mysteries led to a reconsideration of life in 1997 when the mysteries about Venus—past presence of water and the identity of the ultraviolet absorber in the clouds responsible for the dominant deposition of solar energy in the atmosphere—led to new speculations about life in the clouds [22,23,24]. The suggestion that some terrestrial bacteria and proteins show absorption features similar to Venus led to further renewed interest in the possibility of life in the clouds [25,26].

The report of phosphine in the Venus atmosphere [27] has led to more interest and investigations due to it being suggested as a bio-signature [28]. The debate following detection atmosphere has distracted from the primary reasons for suggestions of life on Venus. Recently, Schulze-Makuch [29] present the issues involved, Westall et al. [30] discuss the habitability of Venus [31], and Widemann et al. [32] present a review of planned future missions and the questions they will address. This perspective focuses on the question of possibility of past and present life on Venus with a brief review of the historical context and new findings in the last five years or so.

### The Phosphine Conundrum

With growing interest in habitable exoplanets, Sousa-Silva et al. [28] suggested phosphine as a biogenic signature in search of life on exoplanets. This led Jane Greaves and colleagues to observe Venus to seek any signature of phosphine [27] in the spectra obtained in the mm range using ground-based telescopes. Phosphine was not expected to be present in the Venus atmosphere based on our current (and incomplete) knowledge of the Venus atmospheric chemistry. It is detectable in Earth’s atmosphere due to industrial production as well as natural processes [33]. The detection of phosphine in the atmosphere of Venus [27] has thus been covered in media in the context of a possibility of life on Venus. Such coverage has overshadowed the reasoning behind the original suggestions of origins and survival of life on the surface of Venus in the past and possible evolution to survive in the cloud cover that shrouds the planet today.

The initial detection was swiftly disputed by many [34,35,36,37] with the objections originating in the data analysis procedures used to detect the very low spectral signal of the gas from a bright, extended object such as Venus. This created problems not posed by the deep space astronomical objects that the James Clark Maxwell Telescope (JCMT) and the Atacama Large Millimeter Array (ALMA) commonly observe. The brightness of Venus poses particular difficulties for the ALMA data, as each antenna in the array has a somewhat different characteristic. The single-dish JCMT data presented fewer challenges, and the results were not disputed as vigorously or largely ignored.

The debate about phosphine in the Venus atmosphere involves several issues: (i) is the detection real, (ii) is the detection confused with another species (SO_2_), (iii) what is the altitude level of the reported phosphine, (iv) is the phosphine abiotic or not, and (v) can biology produce phosphine. Although the investigators did not attribute the source of phosphine to life in the Venus atmosphere, the prevailing knowledge of Venus’ atmospheric chemistry appeared to exclude abiotic sources. This has also proved to be controversial, with some [38,39,40] arguing that volcanic sources could produce it, and others arguing just the opposite [41]. Lost in the intense reactions towards the spectroscopic detection and the source of the phosphine was the strong hint of the presence of phosphine in the cloud layer, along with H_2_S, supported by the fractionation product (PH^+^_2_) in the data collected by the PV LNMS [42].

The three main questions—(i) presence of phosphine in the atmosphere at different altitudes, (ii) its origins, and (iii) any extant life—will be settled as new observations are collected from terrestrial telescopes and with future space missions that will need to sample the cloud cover at different altitudes as well as trace constituent profiles in the atmosphere.

## 2. Past Habitability of Venus

The arguments for the possibility of life in the past on Venus have been discussed previously [43,44]. In brief, life requires water, although other solvents have been proposed, including sulfuric acid [45]. We do not know whether early oceans or large water bodies on Earth were acidic (present mean value is 8.2, but turning lower [46] due to increasing atmospheric CO_2_). The current research suggests that over the last 20 million years, the oceans have been slightly alkaline (pH ≈ 8 (https://news-oceanacidification-icc.org/2021/03/19/ocean-acidification-its-lasting-impacts/, accessed on 22 April 2025)). The primary suggestion for past presence of liquid water on Venus comes from the enhanced ratio of deuterium to hydrogen (D/H) in the Venus atmosphere compared with the terrestrial mean ocean water composition [14]. Other mechanisms have also been proposed [47] but have generally not received much support. Secondary suggestions come from the modeling of early climates [48,49], which indicated that liquid water could have survived until as recently as one or two billion years ago, assuming that water condensed onto the surface, inferences of possible minerals that formed under aqueous conditions [50], and geophysical modeling of mantle convection [51,52]. Krissansen-Totton et al. [53], Way et al. [49], Way and Del Genio [54], Warren and Kite [55], and Westall et al. [31] also looked into past habitability from different perspectives and modeling and found Venus habitable in the past under the assumptions and initial conditions used. On the other hand, Constantinou et al. [56] and Turbet et al. [57] found the planet to be too dry with no condensable water and hence inhabitable. Needless to say, there are few data to constrain the modeling assumptions.

### 2.1. Unknowns About Early Venus

Unlike on Earth where fossils and core samples provide some information about past conditions on Earth, Venus presents no such avenue. Thus, only physical similarities with Earth and proximity provide some guidance regarding some of the properties. These include the early rotation rate, nature and extent of the clouds, and planetary albedo. Few means are available to assess the validity of assumed values.

#### 2.1.1. Presence of Liquid Water on Surface

Donahue et al. [14] concluded from the enhanced D/H ratio compared to the Mean Ocean Water composition that Venus had a shallow global ocean, perhaps a few meters thick, by modeling the excess loss of hydrogen to space compared to the heaver deuterium. However, from a modeling study of the evolution of the climate from about a few hundred million years after formation of the crust to present, Turbet et al. [57] arrived at a different conclusion. They argue that Venus never cooled enough to condense water from a steam atmosphere onto the surface to form an ocean [57], as the atmosphere remained “clear” during the day and cloudy during the night, blocking loss of heat and thereby keeping the atmosphere warm. Constantinou et al. [56] also reach a similar conclusion but based on backward modeling of atmospheric chemistry using a general circulation climate model. Not addressed by these studies is the key question of the origin of the water itself—where did it come from?

Water loss on Venus has been estimated from Venus Express data [58,59]. The recent determination of a large variation in the D/H ratio with altitude in the atmosphere [58] provides information about the loss rates on Venus and may shed some light on the likelihood of a liquid water ocean in the past until new measurements of isotopic ratios of noble gases in the Venus atmosphere from the DAVINCI probe [32,59]. It is interesting to note that the Pioneer Venus mass spectrometer data which yielded the high D/H ratio were obtained at an altitude of about 51.3 km [60] only. At higher altitudes, the D/H ratio increases rapidly as determined from the Solar Occultation Infrared Radiometer (SOIR) on Venus Express [58], indicating the complex atmospheric processes active on Venus. With the recent indications that Venus is volcanically active at present [61], it is possible that some water vapor is being emitted along with sulfur dioxide, which may explain the measured altitude profiles of those gases.

Global topography variations on early Venus on regional or smaller scales to allow for lakes and tidal pools should have been present. Such surface pools of water along with hydrothermal vents have been suggested to be the locations for life origins on Earth [62,63].

#### 2.1.2. Rotation Rate

A connection between life and the rotation period is found on Earth through the circadian rhythm [64]. A planet’s rotation period affects the distribution of absorbed solar energy in the atmosphere and surface along with the thickness of the atmosphere and its composition. Lunar tidal effects have slowed the rotation rate of Earth from approximately 6 h to the present-day value of about 24 h. Without a moon of its own, we cannot assess whether Venus also had a much faster rotation period in its infancy.

The rotation rate of Venus (243.0212 ± 0.0006 d) has been measured from Earth-based radar observations during 1988–2017 [65] and differs from the value derived (243.0185 ± 0.0001 d) from the Magellan orbiter radar (1992–1995). Length of day (LoD) variations of as much as 20 min between 2006 and 2020 with a mean rotation period of 243.0226 ± 0.0013 d suggest changes in atmospheric angular momentum with at least ~4% through transfer to the solid planet [66]. Such short-term changes in the rotation rate may be indicative of a far different length of day when Venus formed a crust, if they are assumed as quasi-secular over long periods. However, that is contingent upon atmospheric circulation (surface pressure, wind direction) and surface topography, which have been subject to modification due to volcanic activity and lava flows.

It is possible that the early rotation rate was as short as a few hours, as in the case of Earth. It has been suggested that regardless of the initial rotation rate (and direction) of Venus, it would eventually end up in its present state [67,68,69] of a slow rotation rate with a direction opposite to the orbital motion (i.e., the orbital motion and self-rotation axes are in nearly opposite directions).

With an orbital period of 224.7 days and a rotation period of 243 days, the length of the solar day on Venus (at the surface) is 116.7 days. If the rotation period were much shorter in the past than the present-day value, the solar day would also have been shorter, allowing for larger day–night thermal contrasts (supporting dry and wet conditions on the surface) in a thinner (than present) atmosphere. In a deep atmosphere with zonal circulation, the solar day would vary with altitude depending on the strength and of the flow.

Thus, any life that may have existed on early Venus would have had to adapt to the changes in the rotation rate/solar day. Laboratory experiments with bacteria have shown that such adaptations can take place quickly [70,71]. Microorganism life forms on Venus may have undergone such adaptation and migration from the surface to the habitable clouds in the past. Once the surface became hostile, life forms may have survived in the clouds with a life cycle [26]. If the present-day atmospheric conditions persist on Venus, the settling time for the microorganisms would be long, and the atmospheric circulation could support their presence in the cloud layer [25,26].

## 3. Present Habitability of Venus Atmosphere

Any discussion of life in the clement conditions found in the present-day cloud layer (48–60 km) is contingent upon life having originated or survived on Venus and adapting to the changing conditions and migration to the habitable layer in the clouds [25,26,72]. Origins of life on Earth are still being explored with little consensus, and the possibility that life could have originated on Venus relies on early conditions being similar to those on Earth. The question of origin of life is not addressed here, only whether life could have survived and evolved/adapted is implicit in the discussion.

Possibility of life in the present-day clouds has been discussed previously [22,25,26,44,73,74]. Mogul et al. [75] found that phototrophy is possible in Venus’ clouds. Although the temperatures in the cloud layer (48–70) are within the thermal range within which terrestrial organisms can survive, there are other environmental challenges that any life must overcome. Lacking any means for direct detection of life in the Venus cloud layer, only indirect indicators of life are available. These include chemical disequilibria in the atmosphere and properties of the cloud cover. These and survival challenges are discussed briefly below.

### 3.1. Chemical Dis-Equilibria

Ammonia [15,42,73,76,77,78], molecular oxygen [11,79], methane [80], and phosphine [27] have been reported to be present in the Venus atmosphere below the cloud tops, which indicates chemical disequilibrium based on our current knowledge and available data [53,81,82]. Both biological and abiotic processes can produce these gases and disequilibrium conditions. After the initial dismissal of the ammonia detection, no attempts were made to specifically look for it by the subsequent Venera and Pioneer Venus entry probes. The Pioneer Venus Large Probe Neutral Mass Spectrometer (PV LNMS) data did indicate possible presence of ammonia based on new analysis of the data [42]. Recently, millimeter wave spectral data have been obtained for Venus, and those data also reveal presence of ammonia in the atmosphere as well as phosphine [83]. Following the suggestion that Venus cloud aerosols may contain hydrated ammonia salts, thus lowering the pH, Bains et al. [73] argue that production of ammonia in the Venus clouds makes them habitable.

Phosphine was suggested as a bio-signature of life on exoplanets with hydrogen-rich atmospheres [28], and this led to the search for phosphine on Venus [27], despite its present atmosphere not being rich in hydrogen. Anaerobic bacteria have been found to produce phosphine and methane from sewage sludge through a yet unknown biochemical pathway [84]. It is possible that Venus’ past atmosphere could have been abundant in methane, similar to Earth’s past atmosphere [82,85,86], but not much has been investigated about phosphine on Earth in its past.

Phosphine is not expected to last very long in the present-day Venus atmosphere, so a source is required. Even though the authors did not point to life as a source of the gas, the report faced wide skepticism [34,35,36,37,38,87,88,89,90,91,92,93] and controversy, unfortunately. Subsequent investigations bolster the presence of phosphine without any claims regarding its origins. A few claimed it can be produced geologically [38], while others argued it could not [93,94]. Since then, the new data collected from earth based telescopes by the original authors confirm the presence of phosphine [83].

The presence of methane in the Venus atmosphere remains unconfirmed. While the PV LNMS showed the presence of methane [80], a gas chromatograph (PV GC), the companion instrument on the same probe, did not detect it [11]. New analyses of the PV LNMS may yield more information, but confirmation awaits new measurements to be obtained by the DAVINCI probe in 2031 [59].

Molecular oxygen remains to be confirmed as well, as the PV GC remains the only instrument that has detected it in situ. Above the cloud tops, the upper limit for molecular oxygen is (O_2_/CO_2_) is 10^−7^ [95]. No satisfactory explanation for the simultaneous presence of ammonia and molecular oxygen in the habitable layer has been presented to date.

### 3.2. Environmental Challenges

The main survival challenges to life in the cloud layer (48–72 km) are (i) ionizing radiation, (ii) harmful UV radiation, (iii) water availability, (iv) energy availability, and (v) nutrient availability. Regarding ionizing radiation, a common misperception is that lacking an intrinsic magnetic field, cosmic rays and solar wind will be damaging to life. Unlike on Mars, the thicker atmosphere around Venus leads to an induced magnetosphere, and in the cloud layer, the ionizing and harmful UV radiation are survivable for life [25,43,96,97,98,99,100].

The water availability in the acidic cloud droplets thought to be limiting for life [101] may not be as low as believed according to some new investigations [102,103]. Further, the belief that water may be the only solvent to support life has been challenged by the demonstration that some amino acids and nucleic acids can also survive in concentrated sulfuric acid [45,74,104,105]. The survival of lipids and peptide nucleic acids in concentrated sulfuric acid has been reported as well [106,107], which advances the possibility of life in acidic conditions.

## 4. Life Detection Strategies—Bio-Signatures?

The thinking and approach to detection of life on other worlds has evolved since the first attempts with Viking missions to Mars in 1970s. The two Viking landers each carried three “Life Detection Experiments” and collected data [108]. Of the three, the Labeled Release experiment produced results that have been argued as having detected signatures of life [109] but have not been accepted by the community [110]. With the pause on further data on Mars until the claim that ancient life [111] in the ALH84001 meteorite determined to have come from Mars [112], there were no attempts to investigate the possibility of life on Mars or elsewhere. While the ancient life report was immediately controversial, it shifted the paradigm for search for life from direct detection to finding evidence of liquid water, an essential ingredient for life.

The lessons learned from the search for life have been useful in the approach to detection of life in the clouds of Venus. Various “bio-signatures” have been proposed as evidence for life on other worlds [113,114,115] and have been discussed in “An Astrobiology Strategy for the Search for Life in the Universe” [116]. Schweiterman et al. [117] review remotely detectable signs of life, and Kiang et al. [118] present a summary of the progress in challenges and issues in bio-signature use in detection of life stemming from the community workshop “The Exoplanet Biosignatures Workshop Without Walls” (EBWWW) held in 2016.

Vickers et al. [119] discuss the challenge of determining confidence in the detection of life against a universe of unconceived alternatives and suggest using the framework of developing uncertainties. Smith and Mathis [120] point out the problems with using bio-signatures as a proxy for life: “astrobiology aims to determine the distribution and diversity of life in the universe. But as the word “bio-signature” suggests, what will be detected is not life itself, but an observation implicating living systems. Our limited access to other worlds suggests this observation is more likely to reflect out-of-equilibrium gasses than a writhing octopus. Yet, anything short of a writhing octopus will raise skepticism about what has been detected. Resolving that skepticism requires a theory to delineate processes due to life and those due to abiotic mechanisms. This poses an existential question for life detection: How do astrobiologists plan to detect life on exoplanets via features shared between non-living and living systems?”

Des Marais et al. [113] identify, but do not limit to, the following: (i) Cellular and extracellular morphologies, (ii) Biogenic fabrics in rocks, (iii) Bio-organic molecular structures, (iv) Chirality, (v) Biogenic minerals, (vi) Biogenic stable isotope patterns in minerals and organic compounds, (vii) Atmospheric gases, (viii) Remotely detectable features on planetary surfaces, and (ix) Temporal changes in global planetary properties. Of these, only a few are presently practical for Venus regarding past or present life—stable isotope patterns, atmospheric gases, and temporal changes in global planetary properties.

### 4.1. Atmospheric Composition

The clues to the possible existence of liquid water on Venus in the past may be present in the present composition of its atmosphere. Wordsworth et al. [121] discussed the past N_2_ contents on Earth and Venus and the challenges involved in understanding its past amount even on Earth. The general belief that planetary tropospheres are well mixed in their bulk components is contradicted by Venus, which shows a curious vertical gradient in the abundances of the two main constituents (CO_2_ and N_2_) [122,123]. The high temperatures and pressures in the lower atmosphere near the surface render both CO_2_ and N_2_ into supercritical states. Whether this leads to density separation among the main constituents is uncertain [124,125]. It may also impact the ^15^N/^14^N ratio with altitude, complicating clues about past water.

Beyond ascertaining the make-up of the atmosphere in its bulk constituents (CO_2_ and N_2_) as a function of altitude below 120 km, the abundances of minor constituents (water vapor, sulfur dioxide, sulfuric acid vapor) and trace species (carbon monoxide, ammonia, phosphine, methane, molecular oxygen, hydrogen sulfide, carbonyl sulfide among others), as well as noble gases, are essential for a better understanding of the Venus atmospheric chemistry and supportive environment for life. The planned DAVINCI mission to Venus will deploy a well-instrumented probe into the atmosphere that will make many detailed and more precise measurements below the 70 km altitude than have been obtained to date [59]. While these will improve the data coverage, the global distribution of the constituents will need to be obtained by other future missions.

#### 4.1.1. Minor Constituents: Water Vapor, Sulfur Dioxide, Sulfuric Acid Vapor

The minor constituents of the Venus atmosphere have been sampled only by a few entry probes below about 64 km with low precision [9,11,126,127]. Water vapor, sulfur dioxide, and sulfuric acid vapor have been measured by different instruments at different times, and all show variations with altitude, latitude, and longitude over measurement periods. Greaves et al. suggest some variation in phosphine with latitude and local time from further analysis and new data.

Not much is known about trace constituents (noble gas isotopes, CO, halides, metal oxides, hydrates, and salts in vapor form) abundances and their vertical profiles likely to be present in the Venus atmosphere due to past and present volcanism and meteoritic activity. Many of these are essential for life, e.g., phosphorus [128].

#### 4.1.2. Noble Gas Isotopic Ratios

If there was a liquid water reservoir on the surface, when that was lost either through evaporation and escape to space after hydrolysis or through impacts is an open question. Assuming that the terrestrial planets were formed with similar proportion of nitrogen, then the ^15^N/^14^N isotopic ratio may provide an important constraint of when the primordial ocean may have been lost. The lighter ^14^N would have escaped to space at a higher rate than the heavier ^15^N, thus enriching the atmosphere with ^15^N when the atmosphere was relatively less massive, i.e., before the onset of the runaway greenhouse [129,130]. There is some reason to be cautious about this, as the present-day atmosphere shows a distinct vertical gradient in the bulk composition below about 60 km—the nitrogen content is found to decrease towards the surface from about 5% to 3.5% [11,103,123,131].

Another caution is the role of biology in the nitrogen cycle due to life in hydrothermal vents, which show variations in the nitrogen isotope intake [132] on Earth. Whether this could have affected the atmospheric isotopic ratio is unknown along with whether this could have happened on Venus in its ancient past.

### 4.2. Ultraviolet and Other Absorbers

Ever since the contrasts noticed in visual and photographic observations of Venus in the early twentieth century, the identity of the major absorbers responsible for producing the contrasts through inhomogeneous abundances has been a mystery [21,133,134]. The global cloud cover shows maximum contrasts in reflected sunlight between about 320–400 nm, peaking (40%) at about 365 nm, and weaker contrasts are seen at shorter and longer wavelengths [25,133]. SO_2_ is the only confirmed absorber (below 300 nm) found in the atmosphere, and the identity of other absorbers remains unknown [135,136,137,138,139,140,141].

Several authors have proposed OSSO and its dimers as a possible ultraviolet absorber [142,143,144], but its spectrum and abundances in the Venus atmosphere are not sufficient to explain the observed Venus spectrum [145,146]. As of now, proteins and acidithiobacillus spectra [25] appear to show as good or better match for the Venus spectrum [141] in the 200–500 nm range than any other proposed inorganic absorbers.

While the focus about absorbers has been on the sunlight absorbers, it is worth noting that the near-infrared images of Venus also show contrasts [147,148]. Nightside images of Venus cloud cover (0.8–5.5 μm) also show contrasts in the many CO_2_ windows [149]. The observed structures have been attributed to oxygen airglow (near 1.27 μm, [150,151,152]) and inhomogeneous cloud opacities at other wavelengths. No absorbers have been proposed or detected but some contribution from unknown absorbers cannot be ruled out [25].

### 4.3. Temporal Changes in Global Planetary Properties

**Figure 1:**
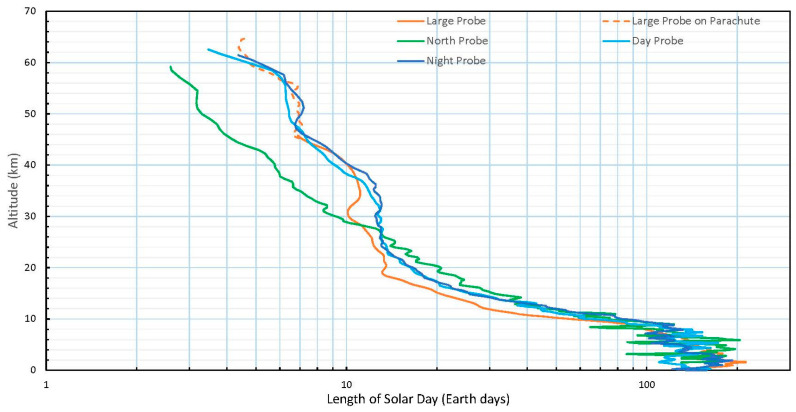
Length of solar day (in Earth days) in the Venus atmosphere from the zonal wind profiles obtained by tracking the Pioneer Venus probes [153]. The cloud top rotation rate determined by following clouds varies between 2.5 days (high latitudes) and ~5 days at the equator and somewhat variable over short and long periods. Tracking of contrasts in near-infrared images (believed to represent ~55–60 km altitude winds) have a period around 5–6 days.

One key property of even microbial life is the presence of a circadian rhythm [64]. Should any life exist in the cloud cover, then it should exhibit such a cycle. In the Venus atmosphere, the length of the solar day varies with altitude due to the dominant atmospheric zonal flow. Figure 1 shows the length of the solar day on present day Venus as a function of altitude.

Monitoring the global cloud cover with multi-spectral imaging from Venus orbiting spacecraft is one means to detect changes in the cloud cover properties. However, reflected solar light observations vary with phase angle, and it can be challenging to separate the temporal variations from phase angle variations. Only in an average sense can these be separated [141]. Spacecrafts in orbit around Venus–Sun L2 Lagrange point offer a solution to monitor the temporal variations in cloud cover with minimal phase angle variations [154]. Akatsuki and Venus Express orbiters have acquired many years of images at multiple wavelengths, and these enable looking for any such periodicities in the reflectivity of the cloud cover as well as determining global scale contrast.

**Figure 2:**
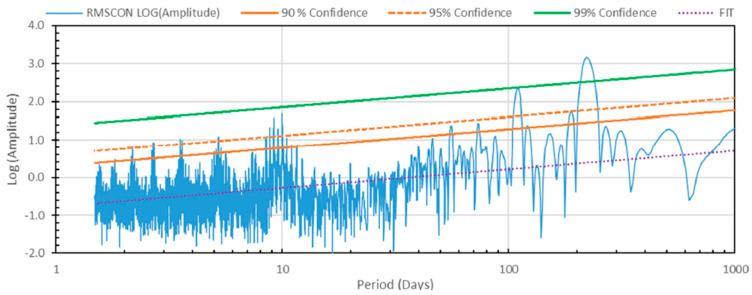
Periodogram contrast (defined as the RMS deviation from Minnaert law for points within ±30° latitude) in the 365 nm images collected by the Akatsuki orbiter during 7 December 2015–30 September 2021 shown as spectral power vs. period. The two dominant peaks (99% confidence) found at 226.5 and 114.7 days correspond to the Venus orbital period and solar day, respectively, and the next group between 8- and 12-day periods (>95% confidence) likely correspond to the Akatsuki orbital period (~10.5 days). The next group includes periods of ~2.1, 2.7, 3.4, and 5.2 days representing the apparent day-night cycle and harmonics in the average contrast in the 365 nm images. The contrasts at 365 nm are believed to be due to absorption in the clouds (48–72 km altitude), and the shorter periods are consistent with the solar day in the cloud layer. Since the images were acquired from a near equatorial orbit with a period varying between 10 and 11 days over the observing duration, it is not possible to separate the orbital variation and a true circadian period which can be possible through imaging from a L2 Lagrange point orbiter [154].

Figure 2 shows the periodicities in the global contrast determined as the root mean square (RMS) deviation from the brightness of the planet predicted by the Minnaert law from 365 nm Akatsuki images [155] obtained during 2016–2018. The strongest peaks reveal the phase angle dependence at the Venus orbital, solar day, and Akatsuki orbiter periods (225, 116, and 10–11 days, respectively), while the other peaks reveal periodicities that correspond to periodic changes in the UV contrasts, to which life forms may contribute with periods matching the length solar day in the cloud layer. It is likely that the atmospheric rotation is also responsible for the periods near 4 days. The existence of such anticipated is of course not an indicator of life, but only corroborative. A short period equatorial orbit is not ideal for determining the circadian period due to the rotation of the atmosphere. A near constant view of the dayside can be obtained from the L2 Lagrange point of the Sun–Venus system, and an orbit around this point would be ideal for continuous day side imaging of Venus [154] to search for the circadian period.

The average cloud top rotation period from tracking 365 nm features varies with latitude (2.5 days at 45 degrees to 5 days at equator) but the dominant contrasts are at low latitudes. The low latitude rotation period over a roughly 12 year period has been found to vary between 4 and 5 days [156] corresponding to a mean zonal speed of 98.6 ms^−1^ and an amplitude of 10 ms^−1^. It is reassuring that the short periods in the contrast periodogram are comparable to the atmospheric rotation period which determines the expected circadian period. But this is not an affirmation of biological circadian activity responsible for the contrasts.

### 4.4. Bio-Organic Molecular Structures

There have been no explicit attempts to detect organic molecules on Venus explicitly, and no organics have been identified in the spectral observations to date. Pathways to organics in the Venus atmosphere were proposed earlier [157], but it is only now that a search will be undertaken. The privately funded Morning Star mission [158] now expected to be launched in 2026 by Rocket Lab (https://www.rocketlabusa.com/missions/upcoming-missions/first-private-mission-to-venus/, accessed on 22 April 2025) will deploy a mini-probe carrying a single instrument to detect the presence of organic molecules [159] using auto-fluorescence. The probe will sample the cloud layer for about three minutes during its rapid descent to the surface. However, the UV contrasts observed in images reveal spatial contrasts, suggesting non-uniform mixing of the absorbers horizontally. If the organics are ubiquitous, there is a possibility that they may be sampled by the probe. The successful return of data from this mission is eagerly anticipated. A negative result in itself may not be indicative of absence of life. The Venus Life finder team has proposed follow on missions after the mini probe mission [160]. Ultimately, cloud aerosol samples may need to be collected for deeper investigation of their composition [161,162,163], but they will need investments in technology for platforms and instruments.

## 5. Remarks

The need for new and improved observations of Venus and its atmosphere/cloud cover are forthcoming. After the anticipated launch of Morning Star mini-probe in 2026, other missions in development may be launched. New missions to be launched by NASA (DAVINCI and VERITAS), ESA (Envision), and India by 2031 will obtain additional new data on Venus and its atmosphere. There may be additional missions from other space agencies and Rocket Lab. Although none of them except the Rocket Lab missions have astrobiology as a science goal, they will nonetheless provide corroborative data on the past and present habitability of Venus. Only the DAVINCI atmospheric entry probe carrying many instruments will make detailed atmospheric measurements below 70 km altitude every 200 m, as it descends to the surface. Two instruments—Venus Mass Spectrometer (VMS) with a wider mass range and higher mass resolution and the Venus Tunable Laser Spectrometer (VTLS)—will sample the atmosphere along the descent path to measure the chemical composition.

The VERITAS, Envision, and ISRO Venus orbiters will map the surface with synthetic aperture radar with an order-of-magnitude better resolution than is available from the Magellan and Venera 15/16 orbiters. They also carry imagers and spectrometers, which have the potential to shed more light on the variable nature of the Venus atmosphere.

The search for life beyond the Earth is motivated by the urge to learn if we are alone in the universe. As many planets around other stars are being discovered, each with its own characteristic atmospheric and stellar environment, with some Earth-like planets deemed habitable, while many others lie in the Venus zone [164] of indeterminate habitability, Venus presents an accessible planet for the search. After the initial excitement of identifying bio-signatures, an era of a cautious approach in the search for life is beginning.

Venus may or may not have had liquid water in its past. If it did, the possibility of life is viable. It is important that we understand our neighbor to assess the possibility of life on Venus like exoplanets. Venus has guarded its mysteries under a thick cloud cover and sampling the clouds is essential to solve this puzzle. With focused, open minded unbiased investigations we may hope to find the answers soon.
